# Spatial analysis of biomineralization associated gene expression from the mantle organ of the pearl oyster *Pinctada maxima*

**DOI:** 10.1186/1471-2164-12-455

**Published:** 2011-09-21

**Authors:** Luke D Gardner, David Mills, Aaron Wiegand, David Leavesley, Abigail Elizur

**Affiliations:** 1 Faculty of Science and Technology, Institute of Health and Biomedical Innovation, Queensland University of Technology, Brisbane, QLD 4000, Australia; 2 Queensland Department of Employment, Economic Development and Innovation, Bribie Island Research Centre, Bribie Island, QLD 4508, Australia; 3 Faculty of Science, Health and Education, University of the Sunshine Coast, Sippy Downs, QLD 4556, Australia

## Abstract

**Background:**

Biomineralization is a process encompassing all mineral containing tissues produced within an organism. One of the most dynamic examples of this process is the formation of the mollusk shell, comprising a variety of crystal phases and microstructures. The organic component incorporated within the shell is said to dictate this architecture. However general understanding of how this process is achieved remains ambiguous. The mantle is a conserved organ involved in shell formation throughout molluscs. Specifically the mantle is thought to be responsible for secreting the protein component of the shell. This study employs molecular approaches to determine the spatial expression of genes within the mantle tissue to further the elucidation of the shell biomineralization.

**Results:**

A microarray platform was custom generated (*PmaxArray *1.0) from the pearl oyster *Pinctada maxima*. *PmaxArray *1.0 consists of 4992 expressed sequence tags (ESTs) originating from mantle tissue. This microarray was used to analyze the spatial expression of ESTs throughout the mantle organ. The mantle was dissected into five discrete regions and analyzed for differential gene expression with *PmaxArray *1.0. Over 2000 ESTs were determined to be differentially expressed among the tissue sections, identifying five major expression regions. *In situ *hybridization validated and further localized the expression for a subset of these ESTs. Comparative sequence similarity analysis of these ESTs revealed a number of the transcripts were novel while others showed significant sequence similarities to previously characterized shell related genes.

**Conclusions:**

This investigation has mapped the spatial distribution for over 2000 ESTs present on *PmaxArray *1.0 with reference to specific locations of the mantle. Expression profile clusters have indicated at least five unique functioning zones in the mantle. Three of these zones are likely involved in shell related activities including formation of nacre, periostracum and calcitic prismatic microstructure. A number of novel and known transcripts have been identified from these clusters. The development of *PmaxArray *1.0, and the spatial map of its ESTs expression in the mantle has begun characterizing the molecular mechanisms linking the organics and inorganics of the molluscan shell.

## Background

For over 500 million years, mollusks have successfully used a variety shells to populate the world over [1]. Due in part to the simple sheer prevalence of mollusks in past and present environments and their variety of shell formation strategies, these organisms represent the current model from which biomineralization is studied. Facilitating the shell formation in molluscs is the mantle organ. Phylum Mollusca is typically classified by an invertebrate unsegmented body, a mantle and a calcareous shell. The latter two are the subject of this investigation. The shell is internally lined by the mantle, composed of a thin sheath of tissue radiating out to the shell margins. In the case of a bivalve this organ is zootomically divided into two regions: the mantle pallial located proximal to the shell hinge, and the mantle edge situated distal to the hinge [2]. The distal mantle is further characterized by enlargement of the sheath at the shell margin into three terminal folds: the outer fold (OF), middle fold (MF), and the inner fold (IF). They are arranged such that OF is closest to the shell and the IF furthest. The main function of the mantle is recognized as the secretion of organic components necessary for shell biomineralization but it also has other purposes [3]. The mantle has a sensory function and can initiate closure of the valves in response to unfavourable environmental conditions [4]. In addition, the mantle also controls inflow of water into the shell's internal chamber responsible for respiratory and filter feeding purposes. These functions are said to be zone-specific in the greater mantle organ, referencing the IF as muscular, MF as sensory, and the OF as secretory in task [3]. Likewise the mantle edge and mantle pallial are considered principally secretory tissues.

To date, the secretory function of the mantle has been the focus of significant research with regard to biomineralization of the shell [1,5,6]. This is especially the case within pearl oyster species, considering pearl cultivation's reliance on mantle tissue. The pearl oyster shell typically consists of an outermost organic layer termed the periostracum, and calcium carbonate oriented in two distinct microlaminates, the outer calcite prismatic layer and the inner aragonite nacreous layer [1]. Evidence in the microstructure of both prismatic and nacreous layers has credited an organic framework as being central to the ordered mineralization [7,8]. As such, the organic component has been the subject of much research devoted to its extraction and characterization [5]. Primarily these investigations have identified a number of matrix proteins, a subset of which have had their corresponding gene sequence determined. Some of those identified include: nacrein [9], MSI60/MSI31 [10], N66/N14 [11] prismalin-14 [12], and caspartin/calprismin [13]. However, many of the proteins remain to be identified, due in part to insolubility, self-aggregation of the molecules or an unusual resistance to temperature, chemicals and enzymes [5,14]. More recently, alternative techniques to identify organic matrix proteins have been employed, including the use of expression cDNA libraries generated from mantle tissue screened with antibodies elicited from unfractionated organic matrix [15]. Although this technique has yielded positive identification of matrix proteins, it is largely inefficient and has meant the expense is inhibitory for most laboratories. Moreover, mantle tissue cDNA libraries have been screened with degenerate primers based on the signal peptide sequences of known proteins [11,16,17]. While this approach has successfully identified a number of organic matrix proteins, this technique is restricted to related proteins, providing little latitude for novel matrix protein detection. Also noteworthy are subtractive cDNA libraries enriched with hundreds of putative organic matrix gene sequences [18,19]. Although the most encompassing method used thus far, subtractive cDNA libraries inherently report only presence or absence of putative organic matrix gene sequences and are incapable of detecting more subtle expression differences. Overall, all the techniques outlined have diverse advantages and limitations however they are still largely inadequate to address the likely complexity of shell biomineralization. A need remains for developing technology by which clusters of genes can be identified and analyzed simultaneously.

Transcriptomics is a recently developing field now readily available for gene discovery and is rapidly being put to use in many novel applications [20]. High-throughput sequencing and EST microarrays facilitate a comprehensive and inclusive experimental approach in which alterations in the state of entire transcriptomes can be simultaneously assayed. This technology has begun to be applied allowing the large scale investigation of gene products expressed in the mantle tissue with reference to biomineralization and other mantle-associated processes [21-23]. Although gene products identified may not necessarily be incorporated in the shell, this technique would circumvent the aforementioned technology limitations. Additionally it should be noted that a transcriptomic approach would not prejudice against gene products potentially involved in biomineralization but not integrated into the shell.

In order to expedite the elucidation of biological processes associated with the mantle organ this investigation has spatially mapped the differential expression of numerous expressed sequence tags (EST) derived from the mantle of *P. maxima *using the custom microarray chip *PmaxArray *1.0 developed for this investigation.

## Results

**Figure 1:**
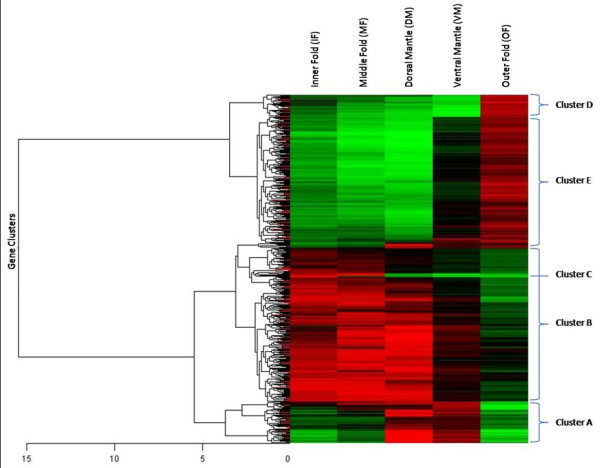
**Heat map displaying ~2000 *P. maxima *ESTs significantly differentially expressed among five discrete spatial regions of the mantle organ: inner fold, middle fold, outer fold, ventral mantle and dorsal mantle**. ESTs are hierarchically clustered according to their spatial expression profile, the largest of which are labelled A to E. The scale of coloration from red to green indicates expression of the EST relative to the control (equal proportion of all conditions) such that green refers to greater relative expression in the control conditions while red signifies greater relative expression in a spatial treatment.

**Figure 2:**
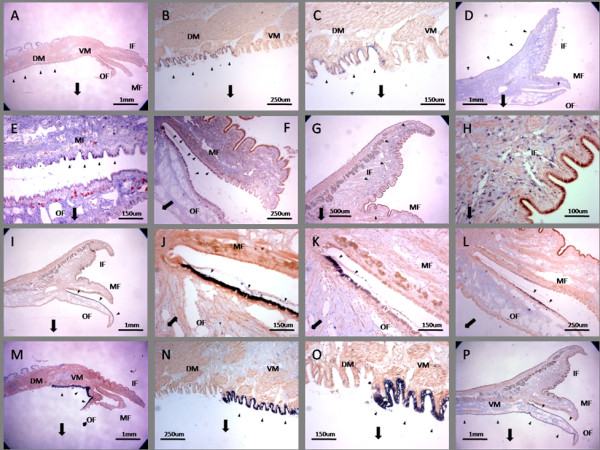
***In situ *expression of 17 *P. maxima *ESTs differentially expressed among mantle regions**. Panels are cross-sectional views of the mantle. Block arrows orient images with respect to the shell. DM = dorsal mantle, VM = ventral mantle, IF = inner fold, MF = middle fold, OF = outer fold. Expression is indicated in dark blue and arrow heads, alternative coloration is background. ESTs displaying similar localization are represented by a single example. (A-C) PM077, PM037 and PM041 [GH280037, GH279997, GH280001] expressed in the outer epithelium of the dorsal mantle, terminating immediately at the ventral mantle region. (D-E) PM316 [JG697411] expressed along the inner epithelium of the mantle and the distal outer epithelial of the middle fold. (F) PM317 [JG697412] expressed proximally in the outer epithelium of the middle fold and the inner epithelium of the outer fold. (G-H) PM315 [JG697410] is discontinuously expressed below the epithelium of the inner fold and middle fold. (I-J) PM233, PM234 and PM235 [GH280193, GH280194, GH280195] expressed along the inner epithelium of the outer fold. (K) PM241 [GH280201] expressed proximally in inner epithelium of the outer fold. (L) PM238 and PM239 [GH280198, GH280199] predominantly expressed mid-way along the inner epithelium of the outer fold. (M-O) PM273, PM268, PM280 and PM281 [GH280233, GH280228, GH280240, GH280241] expressed throughout the outer epithelium of the outer fold and ventral mantle, terminating immediately at dorsal mantle region. (P) PM265 [GH280225] expressed as described for (M-N) in addition to expression in the inner epithelium of the outer fold.

A Kruskal-Wallis test of the data generated from *PmaxArray *1.0 was performed against the five experimental conditions. Outer fold (OF), middle fold (MF), inner fold (IF), ventral mantle (VM) and dorsal mantle (DM) comparisons identified 2012 ESTs of the total 4992 ESTs present on the microarray as statistically differentially expressed in reference to the experimental control (P < 0.001). Hierarchical cluster analysis of these 2012 ESTs grouped them according to similar expression profiles across the conditions. This analysis assisted the selection of four major expression profiles designated clusters A, B, D and E. A sub-cluster of B, termed cluster C was also selected. Clusters of interest were primarily selected based on the likelihood they would be informative in relation to biomineralization characterization. Cluster C was additionally selected due to its extreme difference in expression between the conditions from cluster B as indicated by the colour intensity (Figure 1). A subset of the 2012 ESTs, representing approximately 33% of the corresponding clones, were sequenced and batch blasted against BLASTx (Non-redundant protein sequences nr) and BLASTn (Nucleotide collection nr/nt) databases (Table 1). This subset of the total ESTs identified was deemed sufficient sequence coverage due to redundancy measurements. Many of the smaller cluster's ESTs were sequenced almost in entirety. Sequence alignment software resolved these microarray ESTs to 184 unique sequences. A number of ESTs were selected from each of the five clusters to determine specific local expression in the mantle (Figure 2). These selections were founded on several factors including whether they were either: novel and highly differentially expressed, or share significant homology with annotated genes of interest.

**Table 1:** List of P. maxima ESTs associated with each cluster

	**Transcript Name and Accession No**.	Description of Best Sequence Hit	E-value
	PM078: GH280038	AB032612: *Pinctada maxima *mRNA for N14 matrix protein	0
	PM086: GH280046	DQ352042: *Pinctada margaritifera *calconectin mRNA	1E-177
	PM039: GH279999	EF183520: *Pinctada margaritifera *linkine mRNA	1E-154
	PM072: GH280032	AB032612: *Pinctada maxima *mRNA for N14 matrix protein	1E-137
	PM070: GH280030	D86074: *Pinctada fucata *mRNA for MSI60 protein	4E-55
**Cluster A**	PM076: GH280036	Q3YL58: *Pinctada fucata *mantle gene 8	5E-34
	PM061: GH280021	Q642M8: *Danio rerio *dehydrogenease/reductase	3E-32
	PM074: GH280034	Q86GA3: *Crassostrea gigas *paramyosin protein (fragment).	3E-28
	PM075: GH280035	ABF48089: *Pinctada fucata *EF-hand calcium-binding protein	1E-23
	PM053: GH280013	Q6TL28: *Chlamys farreri*. beta tubulin (fragment)	3E-21
	PM058: GH280018	Q3YL58: *Pinctada fucata *mantle gene 8	2E-18
	PM077: GH280037	Q16UT3: *Aedes aegypti *papilin protein	2E-16
	PM044: GH280004	EDP33798: *Brugia malayi *pancreatic trypsin inhibitor protein	8E-14

	PM306: GH738500	AF547223: *Pinctada fucata *ferritin-like protein mRNA	0
	PM124: GH280084	AF547223: *Pinctada fucata *ferritin-like protein mRNA	1E-152
	PM134: GH280094	AF526224: *Argopecten irradians *ribosomal protein S15	1E-38
	PM113: GH280073	Q27123: *Urechis caupo *cytochrome c oxidase subunit iv	1E-31
	PM120: GH280080	AF379610: *Biomphalaria glabrata *ezrin/radixin/moesin mRNA	2E-31
	PM119: GH280079	AJ561118: *Crassostrea gigas *ribosomal protein S25	3E-26
**Cluster B**	PM114: GH280074	AB076927: *Geloina erosa *cytochrome c oxidase subunit I	7E-25
	PM105: GH280065	ABW90366: *Sipunculus nudus *ribosomal protein L35	1E-20
	PM087: GH280047	AJ563462: *Crassostrea gigas *ribosomal protein L9	5E-20
	PM137: GH280097	AJ563466: *Crassostrea gigas *ribosomal protein L31	2E-18
	PM313: GH738507	A6N9W3: *Ornithodoros parkeri *ribosomal protein S29	2E-15
	PM102: GH280062	Q8M0B7: *Amoebidium parasiticum *cytochrome c oxidase subunit 2	2E-09
	PM092: GH280052	AJ243849: *Sus scrofa *mRNA for glutathione peroxidase	3E-09
	PM307: GH738501	DQ018828: *Argiope versicolor *cytochrome oxidase subunit II	0.0006
	PM104: GH280064	Q7YW83: *Pinctada fucata *ferritin-like protein	0.002

	PM225: GH280185	Q287T6: *Pinctada fucata *tyrosinase	1E-133
	PM233: GH280193	ABO87298: *Pinctada margaritifera *KRMP-5	2E-15
	PM234: GH280194	Q1AGV8: *Pinctada fucata *KRMP-3 protein	3E-15
**Cluster D**	PM236: GH280196	Q1AGV8: *Pinctada fucata *KRMP-3 protein	8E-14
	PM238: GH280198	Q6T6C2: *Theromyzon tessulatum *theromacin	1E-13
	PM235: GH280195	ABP57445: *Pinctada margaritifera *KRMP-7	1E-12
	PM244: GH280204	Q1AGV9: *Pinctada fucata *KRMP-2 protein	2E-10
	PM239: GH280199	Q1AGW0: *Pinctada fucata *KRMP-1 protein	0.0004
	PM226: GH280186	ABP57445: *Pinctada margaritifera *KRMP-7	0.044

	PM270: GH280230	AB429367: *Pinctada maxima *shematrin-3 mRNA	0
	PM274: GH280234	AB429365: *Pinctada maxima *shematrin-1 mRNA	1E-127
	PM264: GH280224	EF160119: *Pinctada margaritifera *shematrin-8 mRNA	1E-112
	PM262: GH280222	AB429365: *Pinctada maxima *shematrin-1 mRNA	1E-103
	PM255: GH280215	AB429365: *Pinctada maxima *shematrin-1 mRNA	5E-90
	PM276: GH280236	EF183519: *Pinctada margaritifera *KRMP-6 mRNA	1E-84
	PM277: GH280237	EF183519: *Pinctada margaritifera *KRMP-6 mRNA	2E-84
	PM273: GH280233	AB429365: *Pinctada maxima *shematrin-1 mRNA	8E-84
	PM275: GH280235	EF183519: *Pinctada margaritifera *KRMP-6 mRNA	5E-82
	PM281: GH280241	EF183519: *Pinctada margaritifera *KRMP-6 mRNA	2E-75
**Cluster E**	PM260: GH280220	EF192240: *Pinctada margaritifera *KRMP-7 mRNA	6E-75
	PM268: GH280228	EF183519: *Pinctada margaritifera *KRMP-6 mRNA	9E-73
	PM280: GH280240	EF183519: *Pinctada margaritifera *KRMP-6 mRNA	1E-69
	PM258: GH280218	EF183519: *Pinctada margaritifera *KRMP-6 mRNA	1E-69
	PM279: GH280239	EF183519: *Pinctada margaritifera *KRMP-6 mRNA	2E-69
	PM278: GH280238	EF183519: *Pinctada margaritifera *KRMP-6 mRNA	3E-67
	PM245: GH280205	AB429365: *Pinctada maxima *shematrin-1 mRNA	4E-63
	PM247: GH280207	AM408910: *Prunus *necrotic ringspot virus coat protein	1E-60
	PM269: GH280229	Q45TK0: *Pinctada fucata *mantle protein 10	4E-53
	PM248: GH280208	AB429365: *Pinctada maxima *shematrin-1 mRNA	6E-41
	PM246: GH280206	EF160120: *Pinctada margaritifera *shematrin-9 mRNA	2E-35
	PM266: GH280226	ABP57445: *Pinctada margaritifera *KRMP-7 protein	6E-17
	PM261: GH280221	ABO87299: *Pinctada margaritifera *KRMP-6 protein	2E-14
	PM272: GH280232	Q1MW94: *Pinctada fucata *shematrin-3 protein	4E-13
	PM254: GH280214	AB429365: *Pinctada maxima *shematrin-1 mRNA	6E-12
	PM265: GH280225	Q27212: *Pseudomicrothorax dubius *articulin protein	0.001

A list of *P. maxima *mantle ESTs with significant sequence similarity to annotated genes and proteins. These ESTs are significantly differentially expressed (P < 0.001) among specific regions of the mantle and have been clustered according to similar expression profiles: cluster A, B, D and E. Description of best sequence hit = highest sequence comparison match of ESTs with Blastx or Blastn search tools, including accession number and brief identification of matching sequence. E-valve = likelihood of random occurrence of sequence match, values approaching zero indicate increasing sequence match significance.

### Cluster A

Cluster A consisted of 225 microarray ESTs typical of the highest relative expression in DM, slightly less expression in VM, and low expression among OF, MF and IF (Figure 1). 197 of the total 225 ESTs were sequenced from which 52 unique sequences were resolved, 21 were contigs and 31 singletons. Putative sequence homologies could only be found for 13 of these ESTs including known shell matrix proteins N14 matrix protein and MSI60 protein. Other noteworthy matches identified are papilin, trypsin inhibitor protein, mantle gene 8 and calconectin (Table 1). A functional domain search of the ESTs significantly similar in sequence with papilin and the trypsin inhibitor protein both revealed tandem Kuntiz trypsin inhibitor domains. The majority of sequences identified from cluster A bear no significant similarity to sequences in public databases, furthermore many of the sequences aligned with poorly described genes and translated proteins. *In situ *hybridization was able to further resolve the localized expression for three ESTs, including; PM077, PM037 and PM041. These ESTs were chosen because they were among the most highly differentially expressed ESTs in cluster A and sequence similarity searches indicated they were novel. The three ESTs were all detected as expressed in the outer epithelium of the dorsal mantle region (Figure 2A-C). Of particular note is that expression of these ESTs is conspicuously absent at what appears the border of the ventral mantle zone and throughout this region.

### Cluster B

Cluster B contained 871 ESTs detailing a relative expression profile as highly expressed in DM, MF, IF, no differential expression in VM, while lowly expressed in the OF in comparison to the control condition (Figure 1). 123 ESTs were randomly selected and sequenced. 68 unique sequences were detected of which 10 resolved as contigs and the remaining 58 singletons. This cluster is principally unannotated; however 15 ESTs are noted for significant sequence similarity to cellular maintenance proteins including: ferritin-like protein, ribosomal proteins, cytochrome oxidase subunits, glutathione peroxidise and radixin (Table 1). *In situ *hybridization was unable to precisely locate any of these sequences in the mantle tissue potentially due to diffuse expression of the target mRNAs impeding *in situ *resolution and/or transcript concentrations being outside the range of detection for the *in situ *hybridization protocol employed in this investigation.

### Cluster C

Cluster C is a small sub-cluster of 22 ESTs within cluster B, characterized by relative high expression present in IF, MF compared to low expression in DM, VM, OF (Figure 1). All 22 ESTs were sequenced, condensing into three contigs and two singletons. Sequence analysis revealed no significant sequence similarity to sequences in the public databases. *In situ *hybridization revealed localized regions of expression of three of the ESTs. PM316 was localised to outer epithelial cells of the MF as well as the inner epithelial cells of the entire mantle organ (Figure 2D-E). PM317 was predominantly expressed in the outer and inner epithelial cells of the ventral sections of folds OF and MF respectively (Figure 2F). PM315 was found to be expressed sub-cutaneously in the IF and MF, specifically appearing interspersed among these regions (Figure 2G-H).

### Cluster D

**Figure 3:**
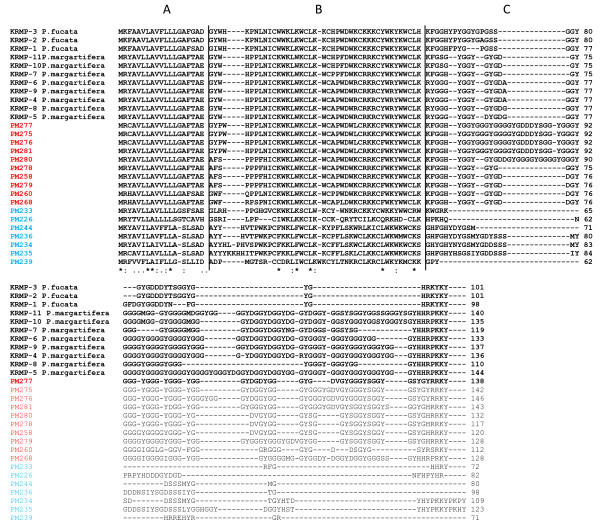
**Alignment of the deduced amino acids of lysine-rich matrix protein family (KRMP)**. ESTs indentified from this investigation as KRMP similar are: PM277, 275, 276, 281, 280, 278, 258, 279, 260, 268, 233, 226, 244, 236, 234, 235 and 239 [GH280237, GH280235, GH280236, GH280241, GH280240, GH280238, GH280218, GH280239, GH280220, GH280228, GH280193, GH280186, GH280204, GH280196, GH280194, GH280195, GH280199]. Previously sequenced KRMP sequences used in the alignment were obtained from the Genbank database http://www.ncbi.nlm.nih.gov: KRMP-1, 2, 3, 4, 5, 6, 7, 8, 9, 10 and 11 [AAZ95763, AAZ95764, AAZ95765, ABO87297, ABO87298, ABO87299, ABP57445, ABP57446, ABP57447, ABP57448, ABP57449]. Sections A, B and C delimited by vertical lines denote the signal peptide, basic region and Gly/Tyr region respectively as set out by Zhang *et al. *[40]. Sequence names labelled red are specific to cluster E, and those labelled light blue are specific to cluster D. Consensus symbols refer to the following: "*" = identical residue in all sequences, ":" = conserved residue substitutions, "." = semi-conserved residue substitution.

Cluster D is represented by 132 ESTs almost exclusively expressed in the OF mantle region (Figure 1). 129 ESTs were sequenced resolving as 21 unique sequences including 12 contigs and nine singletons. Approximately half of these ESTs show significant sequence homologies, the majority of which align with the family of lysine-rich matrix proteins (KRMP) (Figure 3). Additional matches include tyrosinase and thermoacin (Table 1). Alignment of KRMP deduced amino acid sequences with existing protein family members showed these ESTs were significantly divergent from *P. fucata*, *P. margaritifera *and *P. maxima *(cluster E) KRMP's particularly by a general absence of the C-terminal Gly/Tyr region. Of the cluster D homologs only PM244 did not align with all of the 6 cysteine residues present in the basic region (Figure 3). Local spatial expression of six cluster D ESTs was mapped to the mantle. PM233, PM234 and PM235 had similar patterns of expression, detected on the inner epithelium of the outer fold extending the length of the fold (Figure 2I-J). Conversely, PM241 is expressed only in the proximal most inner epithelial cells of the outer fold (Figure 2K), notably absent in expression of the three preceding ESTs (Figure 2J). PM238 and PM239 indicate a further difference in local expression, observed midway along the inner epithelium of the outer fold (Figure 2L).

### Cluster E

Cluster E consists of 762 ESTs showing high levels of expression in OF, no difference to low expression in VM and very low expression in all other conditions in comparison to the control condition (Figure 1). 208 ESTs were sequenced revealing 44 unique sequences, 19 of which are contigs and 25 singletons. Sequence analysis shows 29 of these ESTs have significant similarities to shematrin and KRMP isoforms. Other sequence similarities include a coat protein, mantle protein 10 and articulin (Table 1). The deduced amino acid sequence for the latter two was analyzed for signal peptides and both indicated likely signal peptide sequences. Alignment of KRMP deduced amino acid sequences from cluster E with existing protein family members showed these ESTs all conformed to the typical protein primary structure, particularly with the signal peptide region, basic region and the Gly/Tyr region. Additionally the positions of all six cysteine residues were conserved (Figure 3). *In situ *hybridization indicates spatial expression for five of the cluster's ESTs. PM233, PM237, PM264 and PM268 were detected in the mantle outer epithelium extending from the distil region of the OF into the VM zone after which expression is abruptly absent towards the DM region (Figure 2M-O). Notably, the directly adjacent epithelium is likewise marked by expression of three cluster A ESTs showing a precise border of expression between the ESTs (Figure 2A-C). PM265 has a similar pattern of expression to the four other cluster E ESTs, however, it is additionally expressed in the inner epithelium of the outer fold (Figure 2P).

## Discussion

The molluscan mantle is a thin tissue from which proteins are secreted into the extrapallial fluid; these proteins dictate the animals shell construction and microstructure. As a conserved organ involved in shell formation throughout mollusks, the mantle is an excellent foundation from which to study biomineralization [5]. In this study a *P. maxima *mantle tissue-specific cDNA microarray has been generated termed *PmaxArray *1.0, comprising 4992 cDNA clones derived from the mantle tissue of several *P. maxima *individuals. This tool has provided significant power to interrogate the role of proteins in shell formation.

Microarray analysis has spatially mapped the expression of a number of known and unknown ESTs with reference to specific mantle zones. 2012 ESTs present on *PmaxArray *1.0 were expressed as significantly different to the control condition and approximately one third of those were sequenced and aligned resolving a total of 184 unique ESTs. The majority of those sequences could not be annotated via the Genbank database as no molluscan genome has yet been sequenced, let alone functionally annotated. Other non-model organisms also report a high proportion of unannotated genes [Crustaceans, 60% [24]; Scallop, 73% [25]]. As such, where sequence homologies are absent, functional significance of ESTs identified in this study are interpreted with reference to their pattern of expression (microarray EST differential expression and *in situ *hybridization) and the relevance this bears to mantle associated responsibilities. Five major expression profiles were observed among the mantle zones indicative of specialized molecular functions and ESTs clustering in each of these profiles will be discussed within these groupings.

### Cluster A

The spatial expression profile in cluster A suggests a role associated with the nacreous shell formation of *P. maxima*. Sudo *et al. *[10] along with others [26,27] support this supposition noting a close spatial link between transcript expression in mantle zones and shell microstructure inclusion. Of particular interest within this cluster are PM077 and PM044, as both ESTs possess two tandem KUNTIZ/Bovine pancreatic trypsin domains (KUNTIZ BPT1). PM077 is a significant match to papilin; an extracellular matrix glycoprotein occurring widely from nematodes to humans and known to contain several KUNTIZ domains [28]. Likewise the presence of KUNTIZ domains is expected for PM044 which shares sequence similarity with a pancreatic trypsin inhibitor domain protein. KUNTIZ BPT1 domains are generally regarded as serine protease inhibitors involved in clotting and tissue remodeling [29]. Similarly shell formation is known to involve a number of inhibitory components limiting mineralization. Proteoglycans are one such component, essential to shell formation yet intrinsically inhibit biomineralization [30,31]. The protease inhibiting domains of PM077 and PM044 may act to maintain the viscous silk gel detailed by Adaddi *et al. *[32] as necessary for nacre formation. PM077 is expressed in the DM epithelial cells overlying the nacre microstructure in conjunction with the immediate cessation of expression toward the VM zone and prismatic microstructure. Taken together, tissue localization and sequence homologies suggest that PM077 and possibly PM044 are glycoproteins with inhibitory protease activity specific for nacre formation.

ESTs PM037 and PM041 are unannotated however *in situ *hybridization demonstrated a very specific localization to the epithelium of the DM zone, as already described for PM077. This same distribution of expression is also demonstrated for N14 gene [11,26] and MSI60 gene [10,26] both of which code for nacre matrix proteins. The exclusive expression of these two novel ESTs, PM037 and PM041, suggest a role in nacre formation which along with PM077, are the only reported cases of *in situ *hybridization localizing ESTs to the DM zone since Sudo *et al. *[10] reported MSI60 gene expression.

### Cluster B

This cluster is the largest and most ubiquitous of all the expression profiles identified in this study. ESTs in cluster B display similar expression values across a number of seemingly unrelated mantle tissues. The anatomy and function of the mantle organ is generally considered as follows: OF is secretory (periostracum and shell), MF is sensory, IF is muscular, VM and DM are secretory (shell) [3]. Therefore in the perceived absence of a specialized function uniting these tissues, cluster B most likely represents ESTs involved in general cellular maintenance and regulation rather than shell formation. This proposition is supported by the identification of a number of 'housekeeping' genes (HKGs) not seen in any of the other clusters including cytochrome c oxidase, glutathione peroxidase, ezrin/radixin/moesin binding proteins and ribosomal proteins.

### Cluster C

This cluster is the smallest and contains ESTs which showed no significant similarities with any reported protein or nucleotide sequences. The *in situ *hybridization results for ESTs PM317 and PM316 showed association with the periostracal groove in which the outer epithelium of the MF is included. The main function of the periostracal groove is to secrete a glycocalyx coating forming the periostracum. A glycocalyx is a network of polysaccharides that project from cellular surfaces usually secreted by epithelial cells for a range of adhesion functions. The distil expression of PM316 in the MF outer epithelium indicates this EST may code for a glycoprotein incorporated in the mature stages of the outer glycocalyx coating [33]. Similarly, expression of PM317 in the proximal epithelial cells of the periostracal groove may also code a protein involved in glycocalytic coatings and the stepwise construction of the periostracum.

EST PM315 has a peculiar *in situ *expression pattern in that the transcript is found below the epidermal layer, interspersed throughout the inner region of the MF and the outer region of the IF. Bivalves expose these mantle folds to the external environment [3]. Chemoreceptors, photoreceptors and mechanoreceptors are all usually present in the epidermal layer of these folds in order to elicit closure of the shell valves in response to negative stimulus [34]. Considering PM315 is expressed sub-dermally, it is less likely that this EST has a direct sensory role but rather associated with what appears to be nerve fibres [35], possibly involved in a signal transduction cascade [36].

### Cluster D

The expression profile of cluster D ESTs suggests an exclusive role of these genes in the OF tissue, specifically concerned with the inner epithelia. This epithelium forms the bottom half of the periostracal groove, which is a highly dynamic tissue responsible for formation, maturation and extrusion of the complex periostracum layer. The proteinaceous layer functions to seal the extrapallial space and protect the shell from dissolution as well as serve as an initial matrix for mineralization [33]. *In situ *localization to the inner epithelium of the OF tissue signify a periostracum-related function.

Neuromacin [37] and theromacin [38] are a family of antimicrobial peptides known to occur in a number of invertebrates. These peptides are part of a immediate immune response characterized predominantly by cationic and hydrophobic amino acids [38]. EST PM238 shows a significant sequence similarity to the gene encoding these antimicrobial peptides and its *in situ *expression profile maps it to where the internal periostracum is formed. Cationic and hydrophobic properties of these peptides [38] are synonymous with the characteristics of the periostracum and water insoluble matrix (WISM) of shells [14,39]. Specifically, a scenario for PM238 may be that poly-anionic glycoproteins (shell precursors) bind to cationic peptides in the periostracum, effectively anchoring the hydrophilic macromolecules to the hydrophobic WISM. This in turn facilitates active nucleation sites by which microstructure mineralization occurs.

Lysine-rich matrix protein (KRMP) is a family of proteins seemingly specific to mollusks and shell formation of the prismatic design. Cluster D includes seven ESTs significantly similar to the *KRMP *gene class. Zhang *et al. *[40] first described these proteins noting predominate expression in the inner epithelial cells of the OF and outer epithelium of the mantle edge region. The deduced amino acid sequence includes an N-terminal signal peptide, a lysine-rich basic region potentially interacting with acidic proteins or CO_3_^2-^, and a glycine/tyrosine-rich region considered involved in protein cross-linking via the quinone-tanning process. The expression in the mantle edge region and similarities among the signal peptide of other prismatic shell matrix proteins lead Zhang *et al. *(2006a) to assign a putative prismatic microstructural function to the KRMP family in *P. fucata*. However unlike Zhang's *et al. *[40] observations of dual expression in the periostracal groove and the prismatic mantle region, these *P. maxima *ESTs are exclusively expressed in the OF, a number of which are localized by *in situ *hybridization to the inner epithelium of the fold (PM233, PM234, PM235, PM239), representing the lower half of the periostracal groove. This deviation from Zhang's *et al. *[40] original characterization is potentially explained by sequence analysis. The newly identified *KRMP *members appear to be concatenated versions of *P. fucata KRMP *possessing only the signal peptide and lysine rich region typical of the class. In many of the ESTs the C-terminal region is significantly reduced and/or replaced with serine and aspartic acid residues. The absence of the glycine/tyrosine-rich region suggests that the predicted proteins coded by these ESTs are not quinone-tanned. PM239 is the most divergent of the *KRMP *members and displays a different local expression being present along the middle region of the OF inner epithelium, suggesting a different function, specific to periostracum formation. Unclear however, is whether these ESTs are a novel sub-family of *KRMP *or a species specific evolutionary adaption of *KRMP *in *P. maxima*. The conservation of the lysine-rich region confers the positive charge required to attract and bind acidic glycoproteins necessary for nucleation [41] while expression in the periostracal groove suggests they are incorporated in the periostracum. In summary, the seven *KRMP *homologs in cluster D are considered specifically adapted for periostracal formation in *P. maxima*.

PM241 is a novel transcript expressed in the inner epithelia cells of the OF at the base of the periostracal groove. Periostracum development begins with the formation of the pellicle providing a framework on which coatings of the glycocalyx thicken and develop the periostracum [33]. In bivalves, the pellicle typically originates from a row of basal cells at the bottom of the periostracal groove [42]. The spatial expression of PM241 closely matches the area described for pellicle formation and its deduced sequence is dominated by tyrosine and glycine, typical of a quinone-tanned protein [43,44]. As the pellicle provides the structural backbone on which ensuing glycocalyx coatings mature the periostracum, its formation would be largely concerned with the hardening of the structure.

### Cluster E

ESTs in this cluster are characterised by expression primarily in the OF and VM tissues. The outer epithelia of both these tissues are considered homogenous in function, attributed to prismatic shell formation [10,26,27]. *In situ *hybridization of several cluster E ESTs confirms dual expression in the outer epithelia of the OF and VM, consistent with involvement in prismatic shell formation.

ESTs PM264, PM273, PM274, PM262, PM246, PM255 and PM245 represent the shematrin protein family. While *P. maxima *isoforms for shematrin have already been reported (accession: B1Q4VA) all the ESTs presented here, except PM274, are novel isoforms. Shematrin is a family of glycine-rich shell matrix proteins known to be present in the prismatic microstructure of several pearl oyster species. Yano *et al. *[16] suggests shematrins are framework proteins facilitating calcification of the prismatic microstructure. This investigation maps shematrin isoform PM273 via *in situ *hybridization to the outer epithelium from the tip of the OF to the VM/DM mantle border, parallel with the prismatic/nacreous shell border, adding to the characterization of the shematrin family in relation to the prismatic microstructure.

ESTs PM265 and PM269 show significant sequence homologies with mantle protein 10 and alveolin3 respectively, and both appear to be related to cytoskeletal protein family articulin. Articulins are part of the membrane skeleton of eukaryotic cells stabilizing plasma membranes [45,46]. It is suggested that ESTs PM265 and PM269, function as plateins, a new family of articulins described by Kloetzel *et al. *[47]. Plateins contain modified articulin core domains typical of secreted structural proteins as well as a novel predicted signal peptides detected in intra-alveolar sacs, an extracellular environment. Likewise, PM265 and PM269 also contain predicted signal peptides indicating a secretory pathway and EST PM265 has been detected by *in situ *hybridization specifically to the epithelial cells of both the lower periostracal groove and mantle outer epithelium, in contact with the prismatic shell. These tissues are noted for their secretions reinforcing the secretory pathway of PM265 and PM269. In summary, gene sequence homology of PM265 and PM269 with membrane skeleton proteins, coupled with their differential expression to secretory tissues and detection of signal peptides suggest these ESTs are putative members of a new articulin family, differentiated by extracellular function. These ESTs may encode framework proteins involved in the formation of the prismatic microstructure in *P. maxima *shell.

A functional link between the periostracal groove secretions and prismatic shell formation has previously been suspected based on a structural continuity between the outer periostracum and interprismatic matrices [48]. Zhang *et al. *[40] demonstrated shell matrix protein KRMP as expressed in both secretory tissues. However, the presence of ten KRMP related ESTs found to be expressed specifically in the outer epithelia of the ventral mantle zone three of which were confirmed with *in situ *hybridisation (PM268, PM280, PM281) represent a break from that observed by Zhang *et al. *[40]. The KRMP family has already been discussed in reference to seven EST homologs found to be specific to the periostracal groove of *P. maxima*. The observation of these two separate expression patterns for KRMP related ESTs in the periostrcum and prismatic shell formation mantle regions differs from reports in the related pearl oyster *P. fucata *[40]. In contrast, where KRMP homologues appear to perform dual periostracum/prismatic microstructure roles; *P. maxima *appear to use additional KRMP homologs to accomplish the periostracum related task. This corroborates Jackson's *et al. *[49] supposition that the 'secretome' is a rapidly evolving collection of proteins capable of significant molecular differences in building molluscan shells. In summary, cluster E contains specific KRMP isoforms potentially involved in the prismatic microstructure formation of the *P. maxima *shell. A functional linkage between the periostracum and prismatic shell formation is probable, however the mode by which this occurs is highly adaptable, and unlikely to be conserved among species.

## Conclusions

This investigation has mapped the spatial distribution for over 2000 ESTs present on *PmaxArray *1.0 with reference to specific locations of the mantle. Five major expression profiles were distinguishable from these differentially expressed ESTs (cluster A-E) relating to the examined mantle divisions: dorsal mantle (DM), ventral mantle (VM), inner fold (IF), middle fold (MF) and outer fold (OF). These expression profile clusters have indicated at least five unique functioning zones in the mantle. Three of these zones are considered involved in shell related activities including cluster A's role in nacre formation, cluster D's link to periostracum formation and cluster E's implication in calcitic prismatic microstructure formation. A number of known and novel ESTs have been identified from these clusters. Microarray differential expression, *in situ *expression localization and comparative sequence analysis have begun the task of characterizing novel ESTs identified herein, in addition to further elucidating the functions of previously reported biomineralization related genes. The microarray approach utilized here has alleviated many of the past difficulties plaguing the molluscan biomineralization discipline, however, this method and its' outcomes is in no way seen as a standalone conclusion. Rather, microarray analysis is intended to spearhead preliminary investigations of shell formation targeting ESTs for subsequent in-depth characterization including protein isolation and activity studies.

## Methods

### Microarray development

#### Preparation of RNA

Thirty *P. maxima *animals were collected from several locations on the West Australian and Northern Territory coasts, Australia, courtesy of Paspaley Pearling Company. Animals were immediately anesthetized in 1% propylene phenoxyetol seawater solution until valves were open and non-responsive. This was achieved in less than five minutes. Specimens were then sacrificed and mantle tissue dissected into anterior to posterior strips. Muscle and gill tissue was also sampled. All tissue was stored in RNA*later *(Ambion, Austin, USA). Total RNA was purified from each tissue sample using TRIZOL reagent as recommended by the manufacturer (Invitrogen Life Technologies, Carlsbad, CA, USA). Poly(A)^+ ^RNA was further purified from total RNA when required via Oligotex mRNA Mini Kit as per manufacturer's protocols (Qiagen, Valencia, CA, USA). Concentration and purity of the RNA were determined using a spectrophotometer (GeneQuant Pro, GE Healthcare UK Ltd., Buckinghamshire, England) with 260 and 280 nm readings. RNA quality was assessed for all samples by visualization on a denaturing formaldehyde RNA gel as per the protocol recommended by Qiagen, Valencia, CA, USA) and ethidium bromide staining.

#### cDNA library construction and screening

Two different cDNA library synthesis systems were utilized in order to maximize the diversity of ESTs due to the unknown characteristics of the *P. maxima *mantle tissue.

The first library was created from total RNA pooled from the mantle tissue of 10 individuals using the SMART cDNA library construction kit (Clontech, Mountain View, CA, USA) according to the manufacturer's instructions. Only the final cloning step was modified so that instead of using the λ TriplEx2 vector supplied with the kit, the size fractionated cDNA was ligated into pGEM-T Easy (Promega, Madison, WI, USA) as per manufacturer's instructions, and transformed into XL10 Gold ultracompetent cells (Stratagene, La Jolla, CA, USA) according to the manufacturer's protocol.

The second library produced was a subtractive cDNA library employing the PCR-Select cDNA Subtraction Kit (Clontech, Mountain View, CA, USA). The cDNA synthesized from the mantle poly(A)^+ ^RNA was used as the tester, and cDNA synthesized from muscle poly(A)^+ ^RNA was used as the driver. cDNA fragments were cloned and transformed as the previous mentioned library.

100 clones, randomly selected from each library, were then single extension sequenced by Macrogen (Seoul, Korea) using an Automatic Sequencer 3730 × l. The primer used for sequencing was the 5'SMARTlibPCR primer (5'-AAGCAGTGGTATCAACGCAGAGT-3') a modification of the SMART IV oligonucleotide supplied with the SMART cDNA library construction kit (Clontech, Mountain View, CA, USA). Sequence data was analyzed using Sequencher (Gene Codes Corporation, Ann Arbor, MI, USA) and BLAST http://blast.ncbi.nlm.nih.gov/Blast.cgi to determine EST redundancy.

Upon examination of the 200 clones, from the two cDNA libraries, it was determined redundancies for 16 S ribosomal RNA ESTs were found to be as high as 30% in the SMART cDNA library, while redundancy rates in the subtractive cDNA library were acceptable (< 5%). To remove 16 S ribosomal RNA carrying plasmids from the SMART cDNA library, all of the clones were first screened for the 16 S ribosomal RNA sequence, using a colony hybridization method [50]. Briefly three probes, 500 bp, 344 bp and 300 bp in length were designed from separate regions of the 16 S Ribosomal RNA sequence. These probes were PCR amplified, incorporating Phosphorous^32 ^dATP-labelled radioisotope into the probe's sequence, then hybridized to cDNA library clones that had been fixed to nitrocellulose filters. Following an overnight incubation at 55°C in hybridization buffer (6 × SSC and 1% SDS), the filters were washed twice at 55°C in a solution of 6 × SSC and 0.2% SDS for 30 minutes, sealed within plastic and exposed onto autoradiography films (GE Healthcare UK Ltd.) at -70°C using intensifying screens. The films were then developed according to supplier's instructions.

#### Printing of custom P. maxima mantle cDNA microarrays

4992 unsequenced clones, which had been pre-screened for ribosomal 16 S RNA redundancy, were randomly selected for spotting onto microarray slides. 4224 were selected from the SMART cDNA library and 768 from the subtractive cDNA library. These were grown overnight in LB containing 50 μg/mL ampicillin. The clones were sent to the AgGenomics (Bundoora, Vic, Australia) microarray printing facility. The clones were PCR-amplified using kit-supplied primers (Clontech, Mountain View, CA, USA) and contact-spotted using pins, onto amino silane-coated glass slides, in a 50% DMSO buffer. The 4992 clones were spotted in duplicate on each slide, such that, there was a total of 9984 clones present in two separate grids (technical replicates) on the slides. Known pearl oyster ESTs, which were identified at the initial sequencing stage, including; actin [AF378128], calmodulin [AY341376], myosin [DQ112678], N14 [AB032612] and MSI60 [D86074] were spotted onto the arrays for use as housekeeping and positive controls. In addition, universal reference RNA standard controls (Lucidea, GE Healthcare UK Ltd.) were also spotted onto each array, as were negative control of 50% DMSO (without cDNA). The cDNA was bound to the slide surface by baking and UV-crosslinking.

### Microarray Experimental design

**Figure 4:**
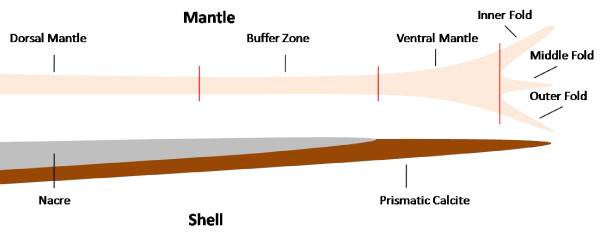
**Schematic diagram depicting a cross-sectional aspect of the *P. maxima *mantle organ and shell *in situ***. Mantle tissues used for the comparative differential gene expression analysis are labelled accordingly: inner fold (IF), middle fold (MF), outer fold (OF), ventral mantle (VM) and dorsal mantle (DM). Red lines denote the approximate position that dissections were made separating the five tissues used in the microarray comparative spatial analysis.

Nine animals were sourced and sacrificed as previously described. Mantle tissue from each animal was dissected under a stereomicroscope into outer fold (OF), middle fold (MF), inner fold (IF), ventral mantle tissue (VM) and dorsal mantle tissue (DM) (Figure 4). Animals selected for dissection had similar shell lengths measuring from the hinge to the opposing shell edge 15 cm (+/- 0.9 cm). This selection aided in standardizing the length of mantle tissue dissected. A strip of mantle tissue from the adductor attachment to the mantle edge along this shell growth axis was dissected from the animal and the fold tissues removed at their junctions. The ventral mantle tissue was dissected immediately adjacent to this junction measuring 0.5 cm in length and 1 cm in width. A buffer zone of 1.5 cm in length was used between the ventral mantle dissection and the following dorsal mantle dissection. The dorsal mantle tissue excised from the animal also measured 0.5 cm in length and 1 cm in width. Total RNA was extracted from these tissues as previously described above and pooled across subjects in order to reduce the effect of biological variation. The total number of subjects and arrays required for the pooled experiment to obtain gene expression estimates and confidence intervals comparable to those obtained from a non-pooled experiment is provided by the formula of Kendziorski *et al. *[51]. The use of nine subjects pooled across a total of three arrays provided the 90% confidence level required. To this effect, equal amounts of total RNA was pooled from the same tissue type from three individuals. This was repeated another two times, totalling nine animals in three separate pools. All the biologically-pooled tissue types were compared against a common reference in which total RNA from all tissues types and all nine animals was equally pooled. Technical variation, which is array-to-array variability, in these microarray experiments was addressed through spot duplication. Two identical grids consisting of each amplified cDNA and including the controls described above were printed onto the left and right sides of each horizontally-orientated array, thus affording spatial separation between duplicate spots, to allow for the normalization of potential hybridization anomalies. As there were five different tissues under investigation, each of which are biologically replicated three times, fifteen *PmaxArray *1.0 slides were consumed. Furthermore each slide has a duplicate technical replicate bringing the final total to 30 arrays for the investigation.

### Microarray hybridization

1 μg of Lucidea universal RNA control (GE Healthcare) was added to 2 μg of pooled total RNA for each tissue type as well as the common reference. The RNA was converted to cDNA then labelled and hybridized to the array using the 3DNA Array 900 MPX expression array detection kit (Genisphere Inc., Hatfield, PA, USA) according to the manufacturer's protocol. Briefly, RNA was reverse transcribed using a random primer combined with an oligo-dT primer. The RNA was then degraded and the cDNA tailed with dTTP followed by ligation to a dendrimer-specific capture oligo (specific for either Cy3 or Cy5). Microarray slides were denatured prior to use by immersion in 95°C MilliQ water for five minutes; the slides were then transferred to 95% ethanol at room temperature for two minutes. Slides were spun dry to reduce streaking at 800 RPM for 2 minutes. The Cy3 and Cy5 "tagged" cDNAs were combined and then hybridized to the array by overnight incubation in a humidity chamber at 65°C using the kit supplied SDS-based buffer and a poly-T-based blocker, as per manufacturer's specifications. The "tagged" cDNA was washed with a series of three SSC-based buffers; the first wash occurred at 65°C for 15 minutes, the other wash steps were carried out at room temperature for 10 minutes each. The slides were spun dry at 800 RPM for two minutes. Fluorescent 3DNA capture reagent (which carries a sequence complementary to the Cy3 and Cy5 tag) was then hybridized to the array using the SDS-based buffer with added Anti-Fade reagent at 65°C for four hours. The fluorescent reagent was then washed as described above for the cDNA hybridization.

### Data Analysis

*PmaxArray *1.0 slides were scanned using a Genepix 4000B scanner (Axon Instruments, Union City, CA, USA) at 10 μm pixel resolution. ImaGene (BioDiscovery Inc., El Segundo, CA, USA) was used to process raw scanner images and create spot intensity reports, while CloneTracker (Biodiscovery Inc.) generated gene ID mapping files and assigned gene identification. Final intensity reports were retrieved as raw spot intensities in tab-delimited files. The data set is deposited in the Gene Expression Omnibus (GEO) database [GSE14303] at the following site: http://www.ncbi.nlm.nih.gov/geo/. Spot intensity reports were imported into data mining software, GeneSight 3.0 (BioDiscovery Inc., El Segundo, CA, USA). Briefly, data was pre-processed and normalized in the following sequence, applying background correction, omitting multiple flagged spots, applying floor correction, omitting low expression spots, calculating ratio values, log-transformation of intensity ratios (base 2), and global LOESS normalization. Ratio data was not normally distributed thus statistical significance among the five tissues were analyzed with a non-parametric, univariate, Kruskal-Wallis test (P < 0.001). Hierarchical cluster analysis was performed among tissues and genes with the Euclidean distance coefficient as distance measure and average linkage.

### Sequence Analysis

ESTs from the *PmaxArray *1.0 identified as significantly significant (P < 0.001) and representing a cluster of interest, were single pass sequenced from their corresponding clones as detailed previously. Vector and poor quality portions of sequence were trimmed and clustered by sequence alignment into singletons and contigs using Sequencher (Gene Codes Corporation, Ann Arbor, MI, USA). These sequences were compared against public protein and nucleotide databases using the BLASTx and BLASTn tools [52] (E value cut-off < 0.01) from the National Center for Biotechnology Information http://blast.ncbi.nlm.nih.gov/Blast.cgi. Where appropriate sequences were analyzed for protein domains, searched against the Pfam database [53] supplied by the Sanger Institute http://pfam.sanger.ac.uk. Deduced amino acid alignments were performed using the ClustalW tool [54] from the European Bioinformatics Institute http://www.ebi.ac.uk. Signal peptides were predicted for sequences using the Signal P 3.0 program [55] from the Center for Biological Sequence Analysis http://www.cbs.dtu.dk/services/SignalP.

### In situ hybridization

RNA anti-sense and sense probes (~400 bp) were generated first by PCR amplifying the EST of interest from cDNA clones using gene-specific primers with T7 and SP6 recognition sequences flanking the 5' end of the primers. 1 μg of the cDNA probe was added to digoxigenin (DIG) RNA-labelling mix (Roche, Penzberg, Germany) as per manufacturer's recommendations for DIG incorporated RNA synthesis. Probes unable to be labelled with DIG were synthesized into unmodified RNA first then non-enzymatically labelled with fluorescein via the Platinum Bright Nucleic Acid Labelling Kit (Kreatech, Amsterdam, Netherlands) according to the manufacturer's protocol. All probes were tested for labelling efficiency using a dot blot technique [50] with the appropriate antibody coupled to alkaline phosphatase.

Mantle tissue was removed from adult *P. maxima *and immediately fixed in 4% paraformaldehyde for four hours. Fixed tissue was dehydrated through an alcohol series and paraffin wax-embedded. Tissue blocks were sectioned to 7 μm increments. Sections were dewaxed in xylene and rehydrated in an alcohol series in preparation for RNA *in situ *hybridization. The technique used RNase-free reagents as described by Braissant and Wahli [56] with some modification. Briefly, rehydrated tissues underwent a 2 × 15 minutes wash in PBS with 0.1% active DEPC; 15 minutes equilibration in 5 × SSC; pre-hybridization, two hours at 50°C, in 50% formamide, 5 × SSC, 40 μg/ml salmon sperm DNA; hybridization 4-40 hours at 50°C, with 400 ng/mL of DIG/FLU labelled probe, in 50% formamide, 5 × SSC, 40 μg/mL salmon sperm DNA; washed 30 minutes in 2 × SSC at room temperature; one hour in 2 × SSC at 60°C; one hour in 0.1 × SSC at 60°C; five minutes equilibration in buffer 1 (Tris 100 mM/NaCl 150 mM, pH 7.5); two hours with anti-DIG/FLU antibody, AP-coupled, diluted 1:1000 in buffer 2 [buffer 1 with 0.5% of Blocking Solution (Roche, Penzberg, Germany)]; washed for 2 × 15 minutes in buffer 1; five minutes equilibration in buffer 3 (Tris 100 mM/NaCl 100 mM, pH 9.5); stained overnight in buffer 3 containing 20 μl NBT/BCIP Stock Solution (Roche, Penzberg, Germany); washed in running tap water for 15 minutes; dehydrated in alcohol series; washed in 95% ethanol for three hours; after which slides were mounted with cover slips.

## Competing interests

The authors declare that they have no competing interests.

## Authors' contributions

LG performed sampling, library preparation, sequencing, bioinformatics analysis of the ESTs, microarray construction, microarray hybridizations, in situ hybridizations, interpretation of the data, and contributed to the overall conception, experimental design of the project and drafting of the manuscript. DM contributed to conception, design, sampling and data interpretation of the experiment. AW contributed to the data interpretation. DL contributed to conception and design of the project. AE contributed to the conception and design of the study, sampling, logistics, data interpretation and drafting of the manuscript. All authors read and approved the final manuscript.
